# A novel strategy for sorafenib-resistant hepatocellular carcinoma: autotaxin Inhibition by PF-8380

**DOI:** 10.1007/s00432-025-06156-3

**Published:** 2025-03-13

**Authors:** Bong Jun Kwak, Jung Hyun Park, Ok-Hee Kim, Dosang Lee, Tae Ho Hong, Sang Chul Lee, Kee-Hwan Kim, Ho Joong Choi, Say-June Kim

**Affiliations:** 1 Department of Surgery, Asan Medical Center, University of Ulsan College of Medicine, Seoul, 05505 Republic of Korea; 2 Department of Surgery, Eunpyeong St. Mary’s Hospital, College of Medicine, The Catholic University of Korea, Seoul, 03312 Republic of Korea; 3 Catholic Central Laboratory of Surgery, Institute of Biomedical Industry, College of Medicine, The Catholic University of Korea, Seoul, 06591 Republic of Korea; 4 Translational Research Team, Surginex Co., Ltd., Seoul, 06591 Republic of Korea; 5 Department of Surgery, Seoul St. Mary’s Hospital, College of Medicine, The Catholic University of Korea, Seoul, 06591 Republic of Korea; 6 Department of Surgery, Daejeon St. Mary’s Hospital, College of Medicine, The Catholic University of Korea, Daejeon, 34943 Republic of Korea; 7 Department of Surgery, College of Medicine, Uijeongbu St. Mary’s Hospital, The Catholic University of Korea, Gyeonggi-do, 11765 Republic of Korea

**Keywords:** Autophagy, Autotaxin inhibitor, Epithelial-mesenchymal transition (EMT), Hepatocellular carcinoma, Sorafenib

## Abstract

By inhibiting the conversion of lysophosphatidylcholine into lysophosphatidic acid, a process pivotal to tumor progression, the autotaxin (ATX) inhibitor PF-8380 offers a new anticancer therapeutic strategy, distinct from the action mechanism of sorafenib. This study explored the potential anticancer effects of the PF-8380 on hepatocellular carcinoma (HCC) cells, especially sorafenib-resistant strains. The investigation included both in vitro and in vivo experiments to evaluate the impact of PF-8380 treatment on epithelial-mesenchymal transition (EMT) and autophagy markers. An orthotopic HCC model served as the in vivo platform. PF-8380 showed a significant reduction in cell viability in both sorafenib-susceptible and resistant HCC cells. It effectively altered EMT by increasing E-cadherin and reducing Snail levels, and inhibited autophagy, as indicated by changes in LC3 and p62 markers. These effects were consistently observed in the orthotopic HCC mouse model, reinforcing PF-8380’s potential as a dual inhibitor of EMT and autophagy in HCC treatment. Our research indicates that PF-8380 could provide substantial therapeutic benefits in the treatment of HCC, even in cases resistant to sorafenib, primarily by suppressing both EMT and autophagy processes.

**Supplementary Information:**

The online version contains supplementary material available at 10.1007/s00432-025-06156-3.

## Introduction

Hepatocellular carcinoma (HCC) is characterized by late-stage detection, underlying cirrhosis, and limited effective treatments, leading to poor prognosis. While various chemotherapeutic agents have been explored, none demonstrated significant survival improvement until the mid-2000s. However, the tyrosine kinase inhibitor sorafenib emerged as a breakthrough in the first-line systemic therapy for advanced HCC. Sorafenib targets VEGFR1, VEGFR2, VEGFR3, PDGFR-β, and RAF-family kinases. In the SHARP phase 3 trial with 602 HCC patients, sorafenib exhibited a mean overall survival benefit of approximately 2 to 3 months compared to placebo (10.7 vs. 7.9 months; *P* < 0.001) (Llovet et al.). Nevertheless, sorafenib often leads to side effects such as diarrhea, hand-foot syndrome, and fatigue, resulting in poor patient compliance. Gastrointestinal events, fatigue, and liver dysfunction were the most common reasons for sorafenib discontinuation (Pang et al.). Furthermore, sorafenib resistance is prevalent, affecting one-quarter of patients in the SHARP trial (Llovet et al.). Consequently, the development of novel or supplementary drugs is crucial for sorafenib-resistant HCC patients, considering the limitations and challenges associated with sorafenib treatment.

Lysophosphatidic acid (LPA) is a simple yet fascinating phospholipid found in nature. It is composed of a single fatty acyl chain, a glycerol backbone, and a free phosphate group. Unlike most other phospholipids, LPA is water-soluble. Despite its simplicity, LPA exists in various structurally diverse forms, suggesting a potential for significant informational content (Mills and Moolenaar 2003; Moolenaar et al. 2004). LPA has been found to induce cell proliferation, migration, and survival, aligning with several characteristics associated with cancer (Mills and Moolenaar). Elevated levels of LPA have been observed in malignant effusions, and its receptors are abnormally expressed in various human cancers (Mills and Moolenaar 2003; Moolenaar et al. 2004; Yung et al. 2014). Autotaxin (ATX) is an enzyme that converts lysophosphatidylcholine (LPC) into lysophosphatidic acid (LPA), playing a role in lipid metabolism and cellular signaling pathways (Erstad et al. 2017; Perrakis and Moolenaar 2014). Significantly, ATX has emerged as a critical factor in facilitating tumor invasion, promoting neovascularization, and facilitating metastasis, underscoring the pivotal role of lysophosphatidic acid (LPA) in cancer progression (Balijepalli et al. 2021; Erstad et al. 2017; Magkrioti et al. 2023). In addition, recent studies suggest that sorafenib treatment, by inhibiting VEGFR and PDGFR, suppresses tumor angiogenesis but concurrently exacerbates hypoxia within the tumor microenvironment. Hypoxia activates hypoxia-inducible factor-1α (HIF-1α), a transcription factor known to induce the expression of ENPP2 (the gene encoding ATX). This upregulation of ATX leads to increased LPA production, which counteracts the effects of sorafenib by promoting cancer cell survival, EMT, and autophagy (Erstad et al. 2017). Thus, inhibition of the ATX-LPA axis may serve as a promising strategy to overcome sorafenib resistance. Notably, the research conducted by Kaffe E. et al. (Kaffe et al. 2017). has underscored the importance of ATX in hepatocyte function, particularly highlighting its role in liver fibrosis and cancer pathogenesis. This body of research has laid the groundwork for further exploration into the therapeutic potential of ATX inhibitors. Several studies indicate an association between increased ATX levels and HCC (Balijepalli et al. 2021; Erstad et al. 2017; Magkrioti et al. 2023; Memet et al. 2018; Wu et al. 2010). However, comprehensive research into the anticancer effects of ATX inhibitors on hepatocellular carcinoma, particularly in sorafenib-resistant HCC, remains insufficient. Our study aims to fill this gap by investigating the anticancer effects of an ATX inhibitor in sorafenib-resistant HCC.

## Materials and methods

### Cell culture and maintenance

Human HCC Huh7 cells, as well as HepG2 hepatoblastoma cells (Cellosaurus cell line Hep-G2, CVCL_0027) and Hep3B cells, were procured from the Korea Cell Line Bank. Both cell lines were cultivated in Dulbecco’s Modified Eagle Medium (DMEM; GibcoBRL) supplemented with 10% fetal bovine serum (Hyclone, UT, USA) and 1% antibiotics (Penicillin-Streptomycin; GibcoBRL, CA, USA). The culture conditions were maintained at 37 °C in a humidified incubator with 5% CO_2_.

Generation of sorafenib-resistant HCC cells.

The wild-type Huh7 cells, designated herein as Huh7-S cells (sorafenib-susceptible Huh7 cells), served as the control group. Sorafenib-resistant Huh7-R HCC (Huh7-R) cells were developed in vitro from Huh7 cells by incrementally increasing sorafenib concentrations. The initial concentration of sorafenib was 1 µM, and it was increased by 0.5 µM every week over a period of six months, culminating at a concentration of 10 µM. Once established, the Huh7-R HCC cells were continually maintained in 1 µM sorafenib.

Cell proliferation assay.

The proliferation of Huh7-W and Huh7-R HCC cells was assessed using the water-soluble tetrazolium salt (WST-1) via the EZ-Cytox Cell Proliferation Assay kit (Itsbio, Seoul, Republic of Korea) according to the manufacturer’s protocol. The cells were seeded in 96-well plates (1 × 104 cells per well) and treated with the ATX Inhibitor PF-8380 for 24 h and 48 h, respectively. Absorbance at 450 nm was measured using a multi-mode reader (Bio-Tek, VT, USA) after the reagent from the EZ-Cytox Cell Proliferation Assay kit was added to each well.

### Wound healing assay

A wound healing assay was performed using Huh7-S and Huh7-R cells seeded in 6-well plates and cultured to full confluence. A straight scratch was made using a 200-µL pipette tip, and detached cells were removed by washing with phosphate-buffered saline (PBS). The cells were then incubated in serum-free medium containing the indicated treatments. Images of the wound area were captured at 0 and 24 h using a phase-contrast microscope. Wound closure was quantified by measuring the difference between the initial wound area at 0 h and the remaining wound area at 24 h. The percentage of wound closure was calculated using the following formula: Wound closure (%) = (Initial wound area– Final wound area) / Initial wound area × 100. Measurements were performed using ImageJ software, and three independent areas per well were analyzed. The experiment was repeated in triplicate to ensure accuracy and reproducibility.

### Western blot analysis

Cell lysates from Huh7-S and Huh7-R HCC cells were prepared using the EzRIPA Lysis kit (ATTO Corporation; Tokyo, Japan). Protein concentration was determined using the Bradford reagent (Bio-Rad, Hercules, CA). Western blot analysis was performed using primary antibodies from Cell Signaling Technology (Beverly, MA) followed by horseradish peroxidase (HRP)-conjugated secondary antibodies from Vector Laboratories (Burlingame, CA). The protein bands were visualized using the Western Blotting Plus Chemiluminescence Reagent (Millipore, Bedford, MA).

### Immunofluorescence and immunohistochemical analysis

Immunofluorescence and immunohistochemical analyses were performed on formalin-fixed, paraffin-embedded tissue sections. After deparaffinization and rehydration, epitope retrieval was carried out. The tissue sections were then stained with antibodies against LC3B, p62, E-cadherin, and Snail (all from Cell Signaling Technology) and examined under a laser-scanning microscope (Eclipse TE300; Nikon, Tokyo, Japan).

### Real-time PCR analysis

Total RNA was extracted using TRIzol reagent (Invitrogen) following the manufacturer’s protocol. Reverse transcription of 1 µg RNA from cells and tissue samples was performed using a RT-premix kit (TOYOBO, Osaka, Japan). Real-time quantitative PCR was performed using SYBR Green with specific primers for E-cadherin, Snail, and human and mouse GAPDH. Relative expression levels were calculated after normalization to the GAPDH gene using the comparative threshold cycle method. Data were presented as the mean ± standard deviation (SD) from three independent experiments.

### Animal studies and experimental design

Orthotopic HCC models were established using five-week-old male BALB/c nude mice procured from Orient Bio, Seongnam, Republic of Korea. Huh7-S and Huh7-R HCC cells (5 × 10^6^) were injected into the liver lobe of each mouse. The animal studies adhered to the guidelines of the Institute for Laboratory Animal Research, Korea (IRB No: CUMC-2020-0211-06). Mice were monitored bi-weekly for weight changes post tumor cell injection. Three weeks after cancer cell injection, mice were randomly assigned to groups (*n* = 5 per group) and treated intraperitoneally with either 10% DMSO in normal saline (control) or PF-8760 (10 mg/kg in 100µL with 10% DMSO in normal saline, 3 times a week) for 30 days. Subsequently, during the laparotomy to confirm tumor formation, the mice were anesthetized for 5 min with a gas mixture of inhaled oxygen (3 mmHg) and nitrous oxide (7 mmHg) administered through a specially designed chamber. Upon completion of the experiment, the mice were euthanized using 100% carbon dioxide gas for a duration of 5 min to facilitate the collection of tissue samples.

### Statistical analysis

All data were analyzed using SPSS 11.0 software (SPSS Inc., Chicago, IL) and expressed as mean ± standard deviation (SD). Statistical comparisons among groups were made using the Kruskal–Wallis test. A probability value (P) less than 0.05 was considered statistically significant.

## Results

### PF-8380 inhibits cell viability in sorafenib-resistant Huh7 cells

**Fig. 1:**
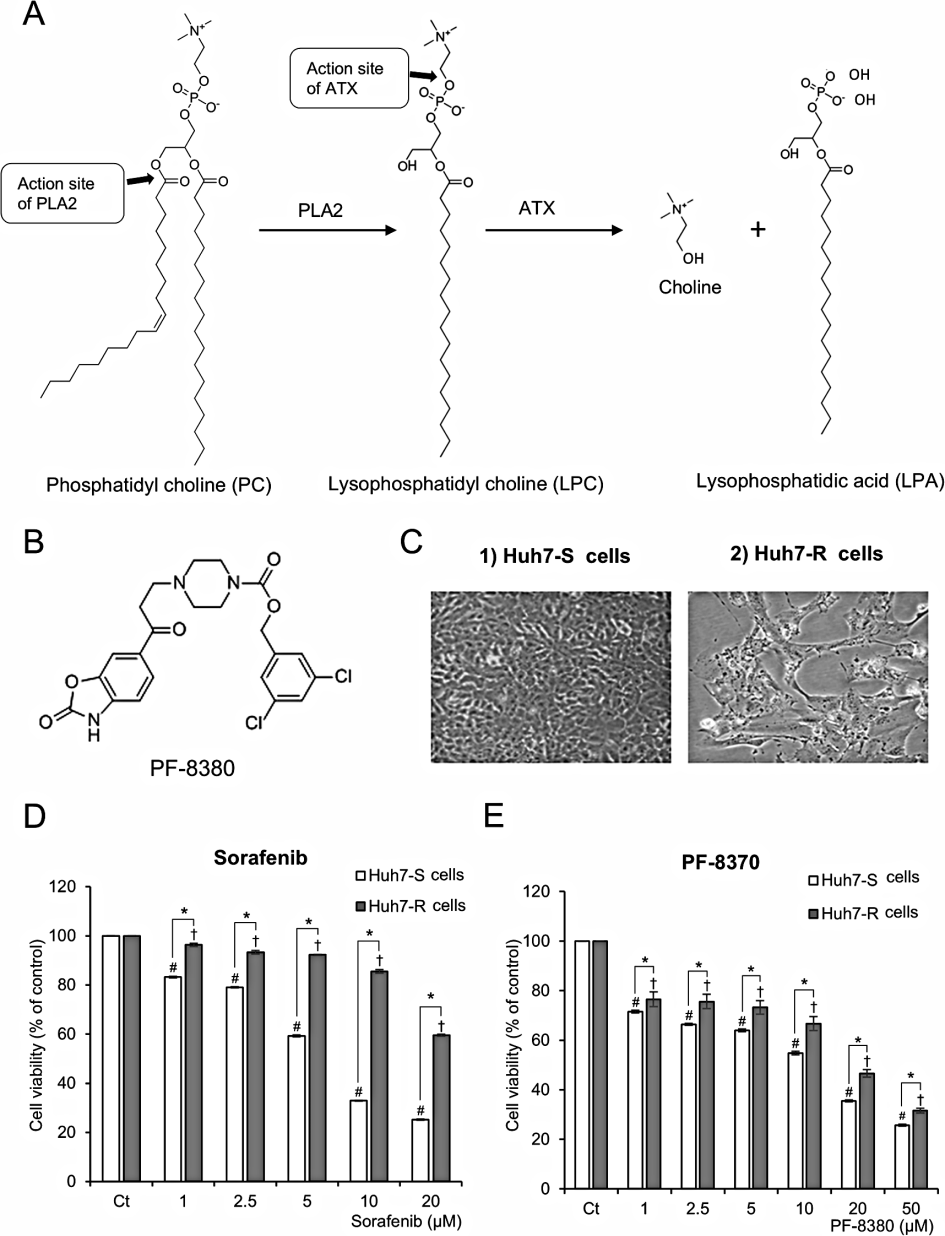
Antiproliferative effects of PF-8380 in Huh7 cells. (**A**) Metabolism of phosphatidyl choline (PC), resulting in generation of lysophophatidyl acid (LPA) by autotoxin (ATX). LPA is recognized for facilitating oncogenic processes, including the promotion of cancer cell proliferation, invasive behavior, and metastatic dissemination. (**B**) Chemical structure of PF-8380, an orally bioavailable piperazinylbenzoxazolone compound acting as an ATX inhibitor. The compound contains two benzene rings, a piperidine ring, an amide group, and a nitrile group. (**C**) Morphological characteristics of sorafenib-susceptible Huh7 (Huh7-S) cells and sorafenib-resistant Huh7 (Huh7-R) cells during culture. These images depict cellular morphology and do not reflect differences in viability. Cell viability upon PF-8380 treatment was separately quantified in Fig. 1E. (**D**) Cell viability of Huh7-S and Huh7-R cells after treatment with increasing concentrations of sorafenib (1–20 µM) for 48 h. Huh7-R cells exhibit higher viability than Huh7-S cells, confirming sorafenib resistance. (**E**) Cell viability of Huh7-S and Huh7-R cells after treatment with increasing concentrations of PF-8380 (1–50 µM) for 48 h. PF-8380 significantly reduces cell viability in a dose-dependent manner in both Huh7-S and Huh7-R cells, indicating its antitumor effect regardless of sorafenib resistance. Values are presented as mean ± SD of three independent experiments. (#*P* < 0.05) compared to the control (Ct) in Huh7-S cells. (†*P* < 0.05) compared to the control (Ct) in Huh7-R cells. (*P < 0.05) between Huh7-S and Huh7-R cells at the same treatment concentration

The generation of lysophosphatidyl choline (LPC) ensues from the hydrolysis of the sn-2 acyl chains of phosphatidylcholine (PC) by phospholipase A2 (ALA2). Autotaxin (ATX) catalyzes a hydrolytic route of LPC, leading to the production of lysophosphatidic acid and choline (Fig. 1A). PF-8380, as an ATX inhibitor, suppresses the production of LPA, which facilitates oncogenic processes, including the promotion of cancer cell proliferation, invasive behavior, and metastatic dissemination. PF-8380 is an orally bioavailable piperazinylbenzoxazolone compound that acts as a substrate competitive and tight-binding inhibitor of autotaxin activity. Its chemical structure consists of two benzene rings, a piperidine ring, an amide group, and a nitrile group (Fig. 1B). The anticancer effects of PF-8380 on hepatocellular carcinoma (HCC) were evaluated in both sorafenib-susceptible Huh7 (Huh7-S) cells and sorafenib-resistant Huh7 (Huh7-R) cells. The morphological characteristics of each cell line during culture are depicted in Fig. 1C. Cell viability was evaluated in Huh7-S and Huh7-R cells after treatment with varying doses of sorafenib (1–20 µM) and PF-8380 (1–50 µM) at 48 h using a viability assay. Huh7-R cells exhibited significantly higher viability than Huh7-S cells following sorafenib treatment, confirming their sorafenib-resistant phenotype (Fig. 1D). At 10 µM sorafenib, Huh7-R cells maintained over 90% viability, whereas Huh7-S cells showed a substantial decrease to below 50%, further supporting the resistance profile of Huh7-R cells. In contrast, PF-8380 treatment for 48 h led to a dose-dependent reduction in cell viability in both Huh7-S and Huh7-R cells (Fig. 1E). Unlike sorafenib, which showed differential effects between the two cell types, PF-8380 effectively reduced viability in both cell lines, indicating its broad antitumor activity irrespective of sorafenib resistance. These findings highlight the potential of PF-8380 as a promising therapeutic strategy for targeting sorafenib-resistant HCC cells by overcoming resistance mechanisms associated with sorafenib treatment. The efficacy of PF8380 extends beyond Huh7 cells, as demonstrated by its capacity to diminish cell viability in both HepG2 and Hep3B cells, with this effect being both dose- and time-responsive (Supplementary Fig. 1).

### PF-8380 inhibits EMT and migration of sorafenib-resistant Huh7 cells

**Fig. 2:**
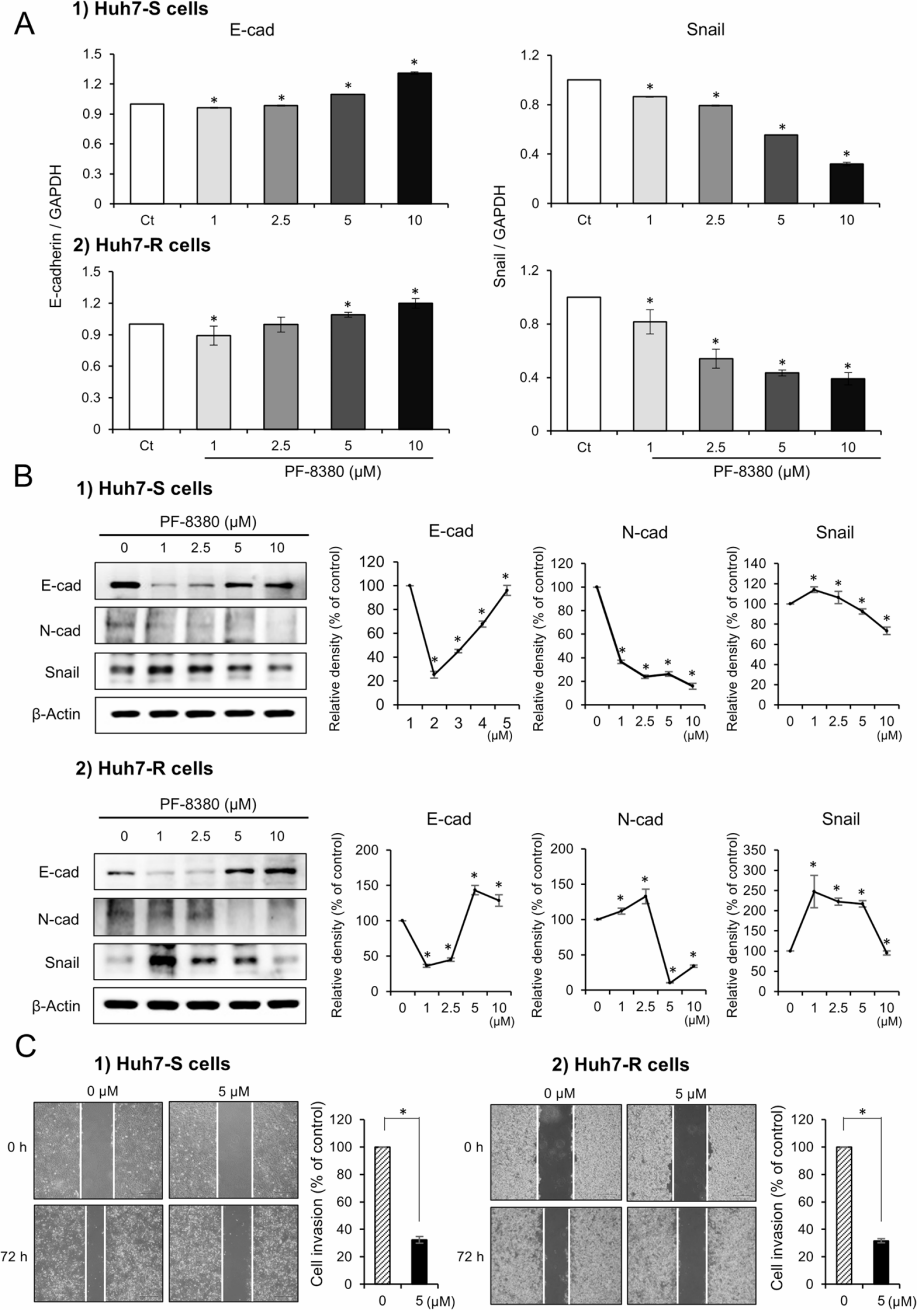
Impact of PF-8380 on EMT and cell migration in sorafenib-resistant Huh7 cells. (**A**) Real-time PCR analysis depicting the alterations in EMT markers post 24-hour treatment with escalating concentrations of PF-8380 (1–10 µM). PF-8380 treatment upregulates the expression of the epithelial marker E-cadherin and downregulates the mesenchymal marker Snail in both Huh7-S and Huh7-R cells. (**B**) Western blot analysis assessing the changes in EMT markers in response to PF-8380 treatment. A dose-dependent effect is observed with an increase in the expression of the epithelial marker E-cadherin, and a decrease in the expression of the mesenchymal markers N-cadherin and Snail in both cell lines. (**C**) Cell migration assay showing the inhibitory effect of PF-8380 on the migratory capacity of both Huh7-S and Huh7-R cells, highlighting its potential role in curtailing cancer cell motility and the propensity for metastasis. Data are represented as mean ± SD of three independent experiments. *P < 0.05

The influence of PF-8380 on EMT was investigated by determining alterations in EMT markers via real-time PCR. Following a 24-hour exposure to escalating concentrations of PF-8380 (1–10 µM), modifications in the EMT markers were detected. Treatment with PF-8380 induced an upregulation of the epithelial marker E-cadherin and a downregulation of the mesenchymal marker Snail in both Huh7-S and Huh7-R cells (*P* < 0.05) (Fig. 2A). Subsequently, the changes in EMT markers were assessed in each cell line through Western blot analysis. PF-8380 exhibited a dose-dependent effect by augmenting the expression of the epithelial marker E-cadherin, while concurrently reducing the expression of the mesenchymal markers N-cadherin and Snail in both cell lines (*P* < 0.05) (Fig. 2B). These observations denote that PF-8380 potentially mitigates EMT in both Huh7-S and Huh7-R cells. Next, a cell migration assay was performed. PF-8380 demonstrated a significant inhibitory effect on the migratory capacity of both cell lines (Fig. 2C), showing to its potential role in curtailing cancer cell motility and the propensity for metastasis.

### PF-8380 exhibits autophagy-inhibitory effects in sorafenib-resistant Huh7 cells

**Fig. 3:**
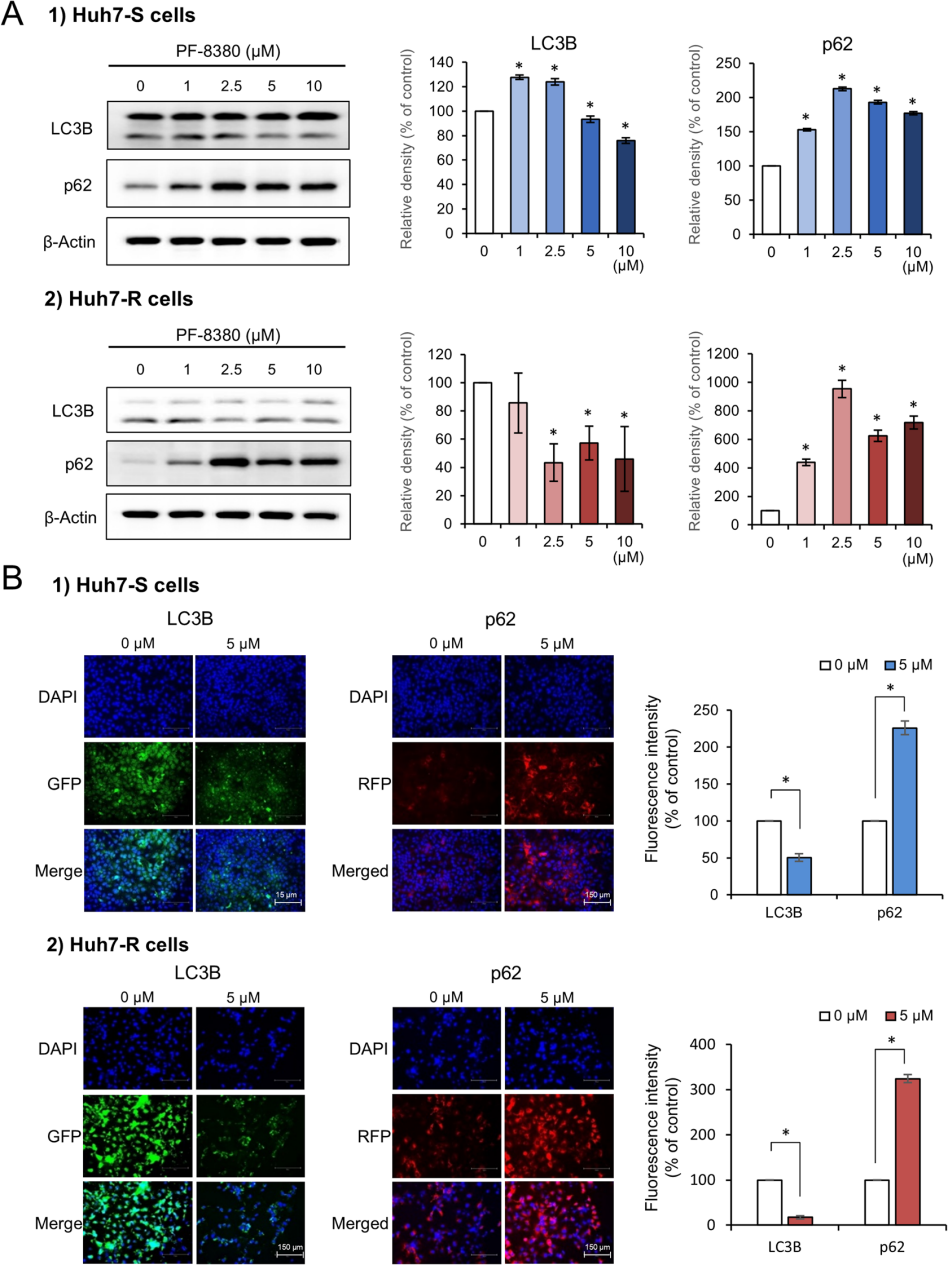
Autophagy inhibition by PF-8380 in sorafenib-resistant Huh7 cells. (**A**) Western blot analysis of autophagy markers LC3 and p62 in Huh7-S and Huh7-R cells following treatment with increasing concentrations of PF-8380 (1–10 µM). A trend of decreasing LC3 and increasing p62 levels is observed in correlation with PF-8380 dose escalation. (**B**) Immunofluorescence of LC3B and p62 in both cell lines pre and post PF-8380 treatment. A marked decrease in LC3B fluorescence and a concurrent increase in p62 is observed post-treatment, indicating an inhibitory effect of PF-8380 on autophagy. Data are represented as mean ± SD of three independent experiments. *P < 0.05

The potential alterations in autophagy markers in response to increasing concentrations of PF-8380 (1–10 µM) were explored in both Huh7-S and Huh7-R cells using Western blot analysis. The examination revealed a discernible trend wherein the autophagy-associated factor LC3 diminished, while p62 escalated in correlation with increasing doses of PF-8380 (Fig. 3A). This reciprocal pattern between LC3 and p62 implicates a potential autophagy-inhibitory role of PF-8380. To further corroborate this hypothesis, immunofluorescence of LC3B and p62 was examined in both cell lines before and after PF-8380 treatment. The post-treatment period marked a significant decrease in LC3B immunofluorescence and a concurrent upsurge in p62 in both cell lines (Fig. 3B), reinforcing the proposition that PF-8380 has the ability to reduce autophagy.

### PF-8380 attenuates EMT in orthotopic sorafenib-resistant HCC mouse model

**Fig. 4:**
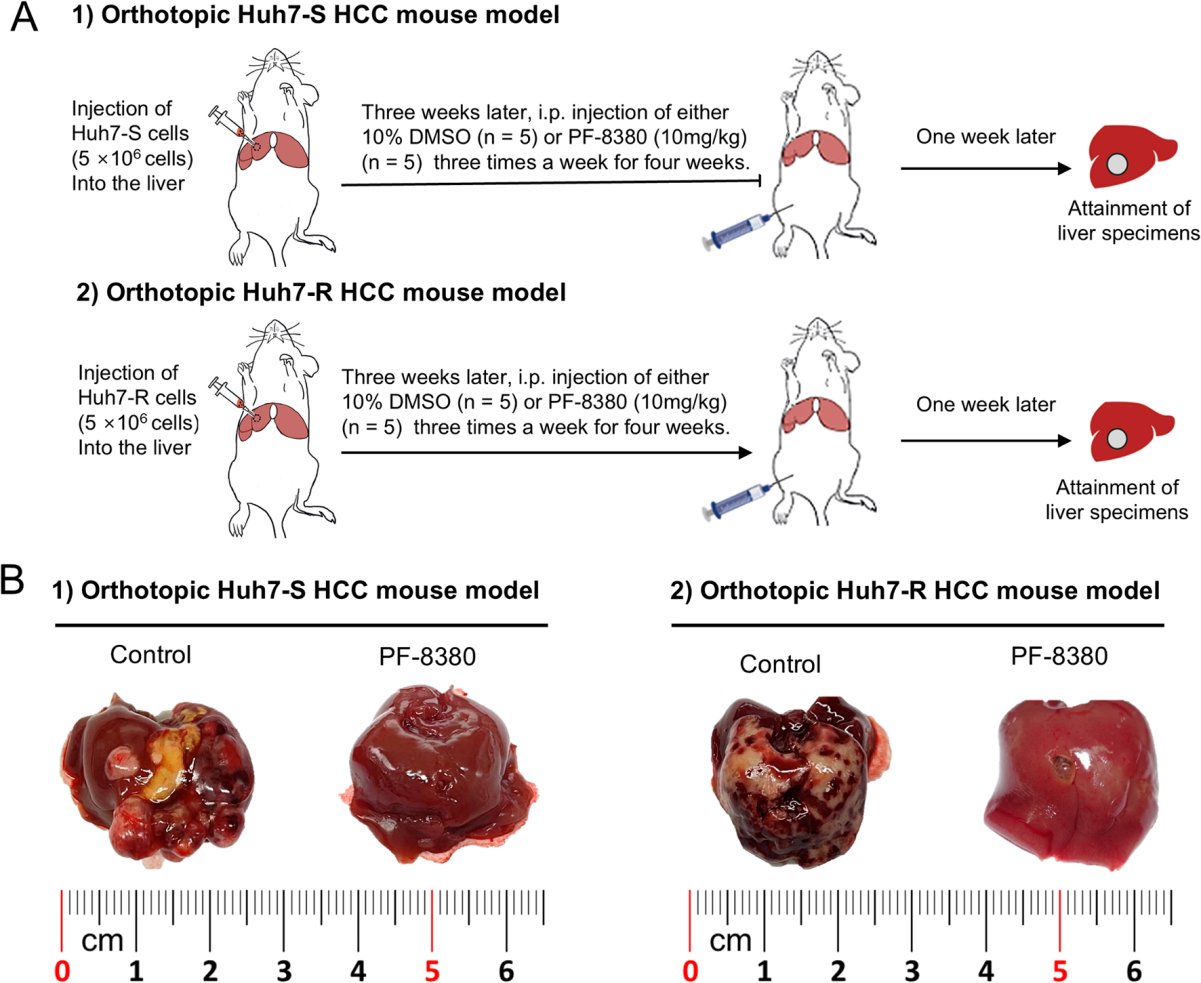
Orthotopic HCC mouse models and their response to PF-8380 treatment. (**A**) Schematic representation of the experimental design. Orthotopic HCC mouse models were established by injecting 5 × 10^6^ Huh7-S and Huh7-R cells into the liver. Three weeks post-tumor implantation, mice were intraperitoneally administered with either 10% DMSO (*n* = 5) or PF-8380 (10 mg/kg) (*n* = 5) thrice weekly over a four-week period. (**B**) Gross morphology of liver specimens collected one week post-treatment. Representative images display the extent of intrahepatic tumor burden in both Huh7-S and Huh7-R mouse models post PF-8380 or DMSO treatment. Notable reduction in tumor cells can be observed in the PF-8380 treated groups

Orthotopic HCC mouse models were established by injecting 5 × 10^6^ Huh7-S and Huh7-R cells into the liver, respectively (Fig. 4A). Three weeks after tumor implantation, mice were injected with either 10% DMSO (*n* = 5) or PF-8380 (10 mg/kg) (*n* = 5) three times a week for four weeks. One week thereafter, mice were euthanized to procure liver mass specimens. All mice in the control group developed tumors, while PF-8380 treatment significantly reduced tumor formation (Fig. 4B). In the control group, both Huh7-S and Huh7-R models exhibited large, irregularly shaped tumors with necrotic regions and invasive growth patterns. In contrast, the PF-8380-treated group showed markedly smaller tumors, with smoother surfaces and reduced necrosis, particularly in the Huh7-R model, where some livers appeared nearly tumor-free. These findings indicate that PF-8380 effectively suppresses tumor progression in both sorafenib-sensitive and sorafenib-resistant HCC models.

**Fig. 5:**
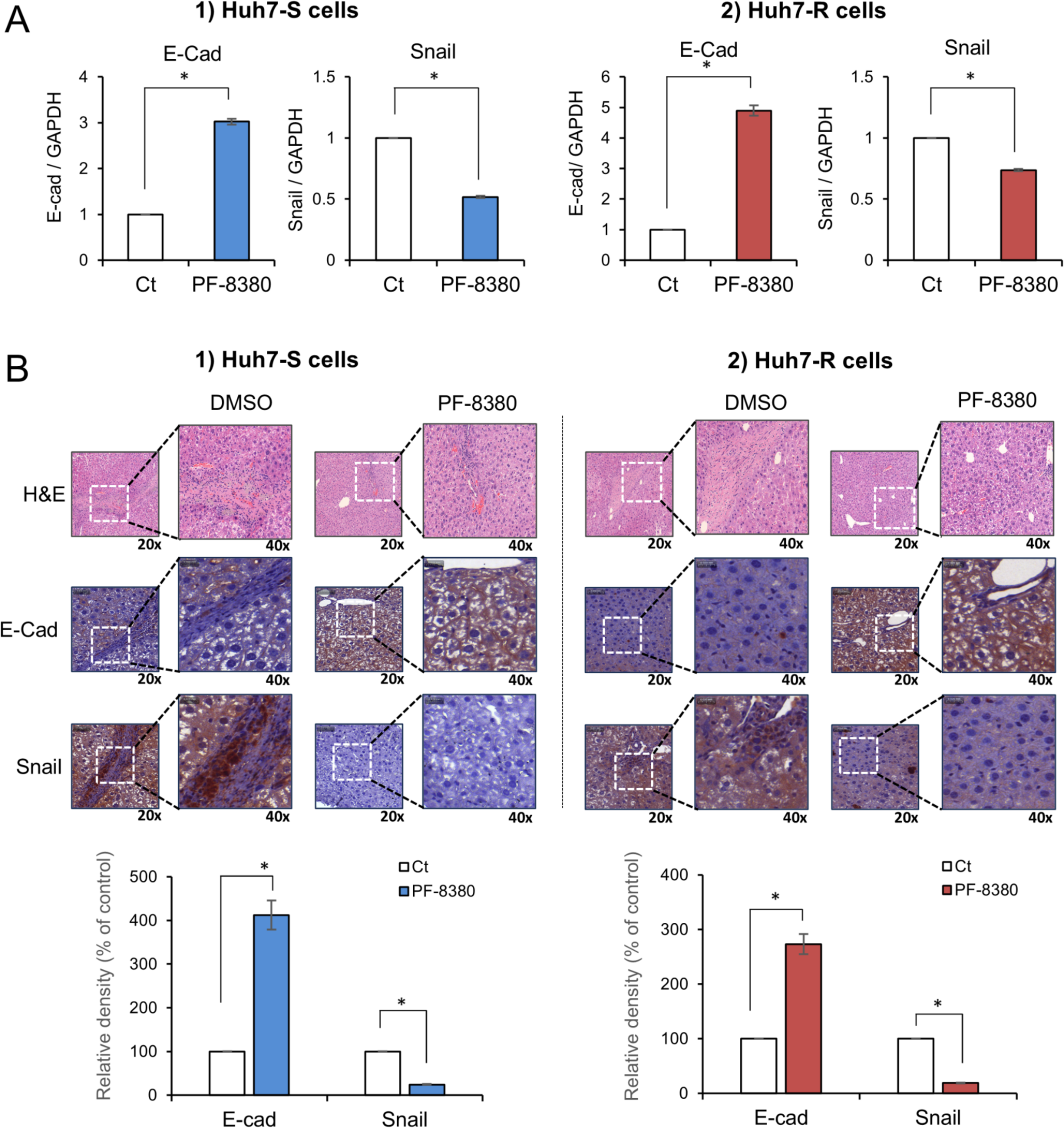
PF-8380 attenuates EMT in orthotopic sorafenib-resistant HCC mouse model. (**A**) Real-time PCR analysis of liver mass specimens revealed that PF-8380 treatment markedly elevated the expression of the epithelial marker, E-cadherin, while concurrently downregulating the expression of the mesenchymal marker, Snail in both Huh7-S and Huh7-R cell-injected cohorts. Data are presented as the mean ± SD, *P < 0.05. (**B**) Immunohistochemical staining of hepatic tumor tissues further confirmed the upregulation of E-cadherin and downregulation of Snail post PF-8380 administration in both Huh7-S and Huh7-R cell-injected groups. Representative images are shown. Scale bar, 50 μm. Quantification data are shown as the mean ± SD of three independent experiments. *P < 0.05

Subsequently, the expression of the EMT-related markers, E-cadherin and Snail, was analyzed in the tumorous hepatic tissues. In both Huh7-S and Huh7-R cell-injected cohorts, PF-8380 treatment was associated with a significant upregulation of the epithelial marker E-cadherin, and a corresponding downregulation of the mesenchymal marker Snail (*P* < 0.05) (Fig. 5A). To further substantiate these findings, we conducted immunohistochemical staining for E-cadherin and Snail using the obtained hepatic tumor tissues. Consistent with the previous results, immunohistochemical staining revealed a significant increase in E-cadherin and a decrease in Snail in both Huh7-S and Huh7-R cell-injected groups post PF-8380 administration (*P* < 0.05) (Fig. 5B). These results collectively suggest that PF-8380 exerts inhibitory effects on EMT.

## Discussion

The study demonstrates that PF-8380 reduces cell viability and inhibits both EMT and autophagy in HCC. PF-8380 suppresses EMT by increasing E-cadherin and decreasing Snail, limiting cancer cell invasion. It also inhibits autophagy, as indicated by reduced LC3 and increased p62, impairing tumor cell survival. In an orthotopic HCC model, PF-8380 significantly reduced EMT, highlighting its potential as a therapeutic option, including for sorafenib-resistant HCC. Additionally, sorafenib treatment has been reported to induce hypoxia, which activates HIF-1α and upregulates ATX expression via ENPP2, leading to increased LPA production. This promotes cancer cell survival and therapy resistance through EMT and autophagy activation (Erstad et al. 2017). PF-8380’s ability to disrupt this ATX-LPA axis suggests its potential to counteract sorafenib resistance mechanisms.

ATX inhibitors, such as PF-8380, have been introduced as potential anticancer therapeutics due to their ability to suppress the production of LPA, a bioactive lipid known to facilitate oncogenic processes including cancer cell proliferation, invasive behavior, and metastatic dissemination (Bhave et al. 2013; Lee et al. 2018). ATX catalyzes the conversion of LPC to LPA, and by inhibiting ATX, PF-8380 effectively reduces LPA levels (Erstad et al. 2017; Perrakis and Moolenaar 2014). ATX inhibitors have been tested in a variety of cancers, including breast cancer (Tang et al. 2020), lung cancer (Bhave et al. 2013), colorectal cancer (Yun 2019), and melanoma (Jankowski 2011). Specifically, in a mouse model of breast cancer, ATX inhibitors were shown to increase the efficacy of radiotherapy and chemotherapy (Tang et al.). ATX inhibitors have also been shown to be effective in reducing the growth of lung cancer cells in vitro and in vivo (Bhave et al.). In addition, ATX inhibitors have been shown to induce apoptosis in melanoma cells (Jankowski). However, while LPA is known to play a substantial role in the pathogenesis of HCC (Balijepalli et al.; Erstad et al.; Magkrioti et al.; Memet et al.; Wu et al.), research investigating the application of ATX inhibitors as anticancer therapeutics for HCC remains markedly limited.

Sorafenib has been the primary therapeutic agent for advanced HCC, but its effectiveness is often limited by severe side effects, including hand-foot skin reaction, diarrhea, and hypertension, which negatively impact patient quality of life and long-term use (Kudo et al. 2018; Pang et al. 2022). More critically, the emergence of sorafenib resistance presents a significant clinical challenge, necessitating the development of novel therapeutic strategies to either replace or complement sorafenib in HCC treatment (Mir et al. 2017; Shen et al. 2010; Yang et al. 2023). In this study, we demonstrated that PF-8380, an ATX inhibitor, effectively suppresses both EMT and autophagy in sorafenib-resistant Huh7 cells. EMT and autophagy are key processes that contribute to tumor progression and therapy resistance, and their inhibition suggests a strong potential for therapeutic intervention. Notably, Huh7-R cells exhibited significant morphological differences from their sorafenib-sensitive counterparts, appearing larger and more elongated. These changes are commonly associated with increased migratory and invasive capabilities, features often observed in cancer stem cells (Li et al. 2020). Given the urgent need for effective treatment options for sorafenib-resistant HCC, our findings indicate that PF-8380 may serve as a promising therapeutic strategy. By targeting ATX and inhibiting the LPA-driven activation of EMT and autophagy, PF-8380 could play a role in reducing tumor aggressiveness and overcoming therapy resistance. This highlights the potential of PF-8380 either as a standalone agent or in combination with existing treatments to improve clinical outcomes in HCC.

**Fig. 6:**
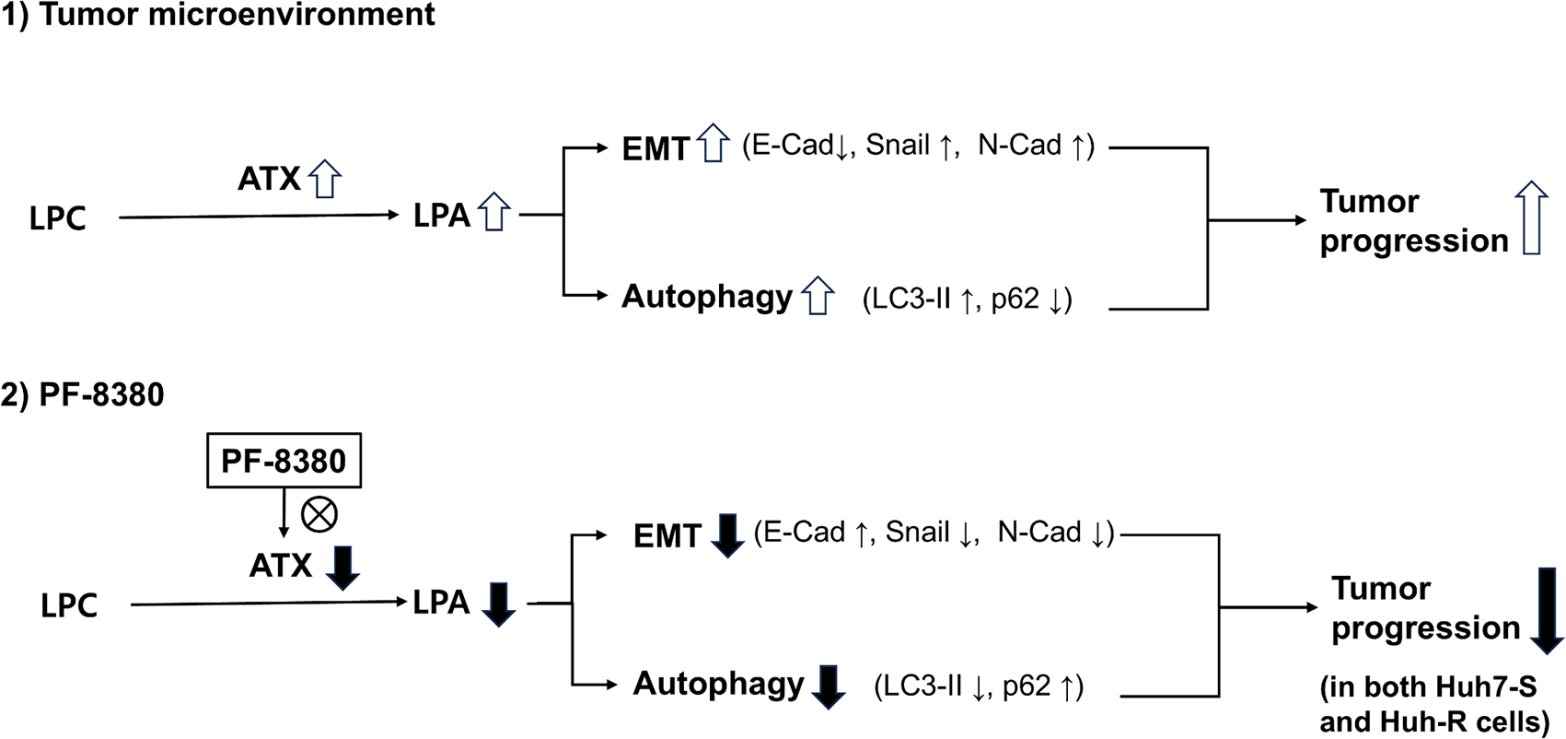
Schematic illustration of the PF-8380-mediated suppression of EMT and autophagy in hepatocellular carcinoma (HCC) cells. (1) In the tumor microenvironment, autotaxin (ATX) catalyzes the conversion of lysophosphatidylcholine (LPC) into lysophosphatidic acid (LPA), leading to the activation of epithelial-mesenchymal transition (EMT) and autophagy. Increased EMT is characterized by decreased E-cadherin (E-Cad) and increased Snail and N-cadherin (N-Cad), while autophagy activation is indicated by increased LC3-II and decreased p62. These processes contribute to tumor progression. (2) PF-8380 inhibits ATX activity, reducing LPA levels, thereby suppressing EMT and autophagy. EMT inhibition is marked by increased E-cadherin and decreased Snail and N-cadherin, while autophagy inhibition is characterized by reduced LC3-II and increased p62. Consequently, tumor progression is suppressed in both sorafenib-sensitive (Huh7-S) and sorafenib-resistant (Huh7-R) HCC cells

PF-8380 suppresses both EMT and autophagy by inhibiting ATX-LPA signaling, which plays a key role in tumor progression (Fig. 6). Although this study did not directly assess the molecular interactions between LPA and these processes, previous research provides a strong rationale for how reduced LPA levels may contribute to EMT and autophagy inhibition. LPA is known to promote EMT by activating key signaling pathways such as PI3K/Akt and MAPK/ERK, which in turn increase the expression of EMT-associated transcription factors such as Snail and Twist (Fukushima et al. 2017). These transcription factors repress E-cadherin and upregulate N-cadherin, facilitating the transition to a mesenchymal phenotype and enhancing tumor invasiveness. Given that PF-8380 reduces LPA production, it is reasonable to infer that this inhibition disrupts LPA-driven EMT signaling, leading to increased E-cadherin expression and decreased Snail and N-cadherin levels, as observed in our study. Similarly, LPA has been reported to promote autophagy, which provides survival advantages to tumor cells under stress conditions. One of the mechanisms involves Beclin-1 activation, which facilitates the formation of the PI3KIII complex, a key component of autophagosome formation (Chen et al. 2017; Koike et al. 2009). Additionally, LPA has been shown to suppress mTOR, a well-known inhibitor of autophagy, further enhancing autophagic activity (Zhang et al. 2014). Since PF-8380 inhibits ATX and reduces LPA levels, it likely interferes with these autophagy-promoting mechanisms, contributing to the observed decrease in LC3-II and increase in p62, indicative of autophagy inhibition. Taken together, these findings suggest that LPA reduction through ATX inhibition disrupts EMT and autophagy-promoting pathways, thereby contributing to tumor suppression. While further studies are needed to fully elucidate these mechanisms, our results strongly indicate that PF-8380 effectively modulates these key oncogenic processes by targeting ATX-LPA signaling.

Autotaxin plays significant roles in various conditions, spanning both nonmalignant and malignant diseases. Nonmalignant conditions include fibrosis and inflammatory disorders, while its role in malignancies primarily relates to cancer progression and metastasis. This study specifically investigated the effects of the autotaxin inhibitor PF-8380 on sorafenib-resistant HCC. However, the potential applications of PF-8380 could extend to other diseases where autotaxin is implicated. Future research is recommended to explore the broader therapeutic uses of autotaxin inhibitors like PF-8380 in these contexts. Such studies could provide valuable insights into the versatility and efficacy of PF-8380 across a spectrum of autotaxin-related diseases.

In conclusion, this study has unveiled the potential of PF-8380, an ATX inhibitor, as a viable therapeutic strategy for HCC, particularly in cases showing resistance to the standard treatment with sorafenib. PF-8380 demonstrated significant inhibitory effects on both EMT and autophagy, two critical processes that drive the pathogenesis and progression of HCC. This reinforces the potential of ATX inhibitors as significant contributors to the arsenal of anticancer therapeutics for HCC. However, it is important to acknowledge that the study was conducted in preclinical models and the results, though promising, need to be validated in a clinical setting to determine their translational potential. Further research is warranted to delve deeper into the underlying mechanisms of action and to optimize the therapeutic application of PF-8380 in the management of HCC.

## Electronic supplementary material

Below is the link to the electronic supplementary material.

Supplementary Material 1

## Data Availability

No datasets were generated or analysed during the current study.
