# Supplementary material for: A novel strategy for sorafenib-resistant hepatocellular carcinoma: autotaxin Inhibition by PF-8380
Source: J Cancer Res Clin Oncol. 2025 Mar 13;151(3):110. doi: 10.1007/s00432-025-06156-3 (PMC11906571; doi:10.1007/s00432-025-06156-3)

Supplementary Figure 1. Effects of MF8380 on the cell viability of HepG2 and Hep3B cells. PF8380 reduced cell viability in HepG2 (A) and Hep3B (B) cells in a dose- and time-dependent manner. Values are presented as mean ± SEM of three independent experiments. * P < 0.05.


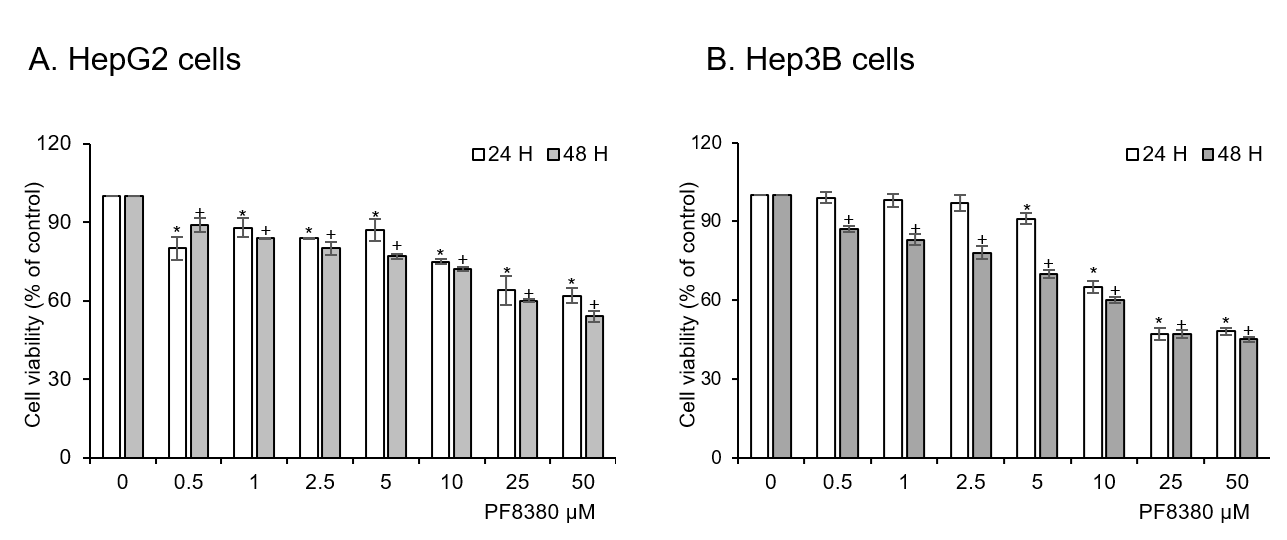

Supplement: Supplementary file 1 — Supplementary Material 1 [file 432_2025_6156_MOESM1_ESM.docx]
